# Targeting danger molecules in tendinopathy: the HMGB1/TLR4 axis

**DOI:** 10.1136/rmdopen-2017-000456

**Published:** 2017-07-28

**Authors:** Moeed Akbar, Derek S Gilchrist, Susan M Kitson, Briana Nelis, Lindsay A N Crowe, Emma Garcia-Melchor, James H Reilly, Shauna C Kerr, George A C Murrell, Iain B McInnes, Neal L Millar

**Affiliations:** 1 Institute of Infection, Immunity and Inflammation, College of Medicine, Veterinary and Life Sciences, University of Glasgow, Glasgow, Scotland, UK; 2 Department of Orthopaedic Surgery, Orthopaedic Research Institute, St George Hospital Campus, University of New South Wales, Sydney, New South Wales, Australia

**Keywords:** inflammation, tendinopathy, cytokines, HMGB1, TLR4, translational

## Abstract

**Objectives:**

To seek evidence of the danger molecule, high-mobility group protein B1 (HMGB1) expression in human tendinopathy and thereafter, to explore mechanisms where HMGB1 may regulate inflammatory mediators and matrix regulation in human tendinopathy.

**Methods:**

Torn supraspinatus tendon (established pathology) and matched intact subscapularis tendon (representing ‘early pathology’) biopsies were collected from patients undergoing arthroscopic shoulder surgery. Control samples of subscapularis tendon were collected from patients undergoing arthroscopic stabilisation surgery. Markers of inflammation and HMGB1 were quantified by reverse transcriptase PCR (RT-PCR) and immunohistochemistry. Human tendon-derived primary cells were derived from hamstring tendon tissue obtained during hamstring tendon anterior cruciate ligament reconstruction and used through passage 3. In vitro effects of recombinant HMGB1 on tenocyte matrix and inflammatory potential were measured using quantitative RT-PCR, ELISA and immunohistochemistry staining.

**Results:**

Tendinopathic tissues demonstrated significantly increased levels of the danger molecule HMGB1 compared with control tissues with early tendinopathy tissue showing the greatest expression. The addition of recombinant human HMGB1 to tenocytes led to significant increase in expression of a number of inflammatory mediators, including interleukin 1 beta (IL-1β), IL-6, IL-33, CCL2 and CXCL12, in vitro. Further analysis demonstrated rhHMGB1 treatment resulted in increased expression of genes involved in matrix remodelling. Significant increases were observed in Col3, Tenascin-C and Decorin. Moreover, blocking HMGB1 signalling via toll-like receptor 4 (TLR4) silencing reversed these key inflammatory and matrix changes.

**Conclusion:**

HMGB1 is present in human tendinopathy and can regulate inflammatory cytokines and matrix changes. We propose HMGB1 as a mediator driving the inflammatory/matrix crosstalk and manipulation of the HMGB1/TLR4 axis may offer novel therapeutic approaches targeting inflammatory mechanisms in the management of human tendon disorders.

**Key messages**

**What is already known about this subject?**

- Tendinopathy is a complex multifaceted tendon pathology with molecular evidence suggesting a role for inflammatory mechanisms in disease. The pathological process of tendinopathy implicating repetitive microtrauma/stress lends itself as a plausible alarmin-mediated pathology.

**What does this study add?**

- This study shows that the alarmin, high-mobility group protein B1 (HMGB1), is present in human tendinopathy and can regulate inflammatory cytokines and matrix changes through toll-like receptor 4 (TLR4) signalling. The adds further evidence to the key role of inflammatory mechanims in tendon homeostasis.

**How might this impact on clinical practice?**

- Manipulation of the HMGB1/TLR4 axis may offer novel therapeutic approaches targeting inflammatory mechanisms in the management of human tendon disorders.

## Introduction

Overuse tendon injuries, namely tendinopathies, pose a significant, highly prevalent problem in rheumatological medicine with the diagnosis and management of shoulder tendon injuries alone amounting to an annual cost of $3 billion to the US healthcare system highlighting the huge burden of disease.^1^ The intrinsic pathogenetic mechanisms underlying the development of tendinopathies are largely unknown; however, inflammatory mechanisms have recently been implicated functionally in several model systems.^2^ Molecular evidence suggests that many of the key inflammatory interactions occur in the early stages (acute/subacute) of repetitive tendon microtrauma when patients can be totally asymptomatic.^3^ At these early stages, changes in tissue microenvironments and crosstalk with the innate immune system interact at a crossroads between reparative versus degenerative ‘inflammatory’ healing. Additional evidence suggests that repetitive biomechanical stress and its associated damage in stromal tissues play a key role in the immune systems response to regeneration.^4^ Thus, the pathological process of tendinopathy implicating repetitive microtrauma/stress and dysregulated matrix remodelling lends itself as a plausible alarmin-mediated pathology.

Alarmins, also known as danger signals, are members of the damage-associated molecular group of proteins (DAMP) that are rapidly released into the extracellular compartment during tissue damage. High-mobility group protein B1 (HMGB1) is a widely expressed, highly conserved nuclear protein involved in transcriptional regulation playing principal roles in a variety of inflammatory responses and diseases.^5^ HMGB1 acts as a DAMP after its release, which can occur passively from dead cells or actively by secretion from activated immune cells, and other cell types under stress.^6^ HMGB1 signals through a family of receptors including receptor for advanced glycation endproducts (RAGE), toll-like receptor 2 (TLR2), TLR4, TLR9 and CD24-Siglec-10 while also forming heterocomplexes with interleukin 1 beta (IL-1ß), CXCL12 or lipopolysaccharide(LPS).^7 8^ Importantly, stress conditions (hypoxia, mechanical) which are considered key to the onset and perpetuation of tendinopathy^9^ result in HMGB1 release. Extracellular HMGB1 induces several responses, including the elaboration of proinflammatory cytokines, cell proliferation and stromal cell matrix responses.^10^ Emerging evidence indicates that HMGB1 contributes to many diseases with dysregulated matrix responses such as rheumatoid arthritis (RA), liver fibrosis and progressive pulmonary fibrosis^11^ and HMGB1 binding and signalling through TLR4 mediate cytokine tissue injury. Moreover, agents targeting HMGB1 have delivered promising in vitro and in vivo data^12^ particularly altering matrix regulation, suggesting that HMGB1 may offer therapeutic utility.

One of the major limitations of human studies is that tendon biopsies are usually obtained when patients are symptomatic and therefore biopsy material is likely to represent chronic, rather than early phase processes. We previously suggested that matched subscapularis tendon from patients with full thickness rotator cuff tears may be a model of early human tendinopathy based on histological appearances and significantly increased levels of cytokines and apoptotic markers in these tissues.^13^ The purpose of this study was to formally assess the expression of HMGB1 within this human model of tendinopathy and thereafter explore the mechanistic activities of HMBG1 on inflammation and matrix production in tenocytes in vitro.

## Methods

### Human model of tendinopathy

All procedures and protocols were approved by the Ethics Committee under approval number No. 99/101 with informed consent obtained and carried out in accordance with standard operative procedures. Eight supraspinatus tendon samples were collected from patients with rotator cuff tears undergoing shoulder surgery. The mean age of the rotator cuff ruptured patients was 50 years (range, 35–64 years) (table 1)—the mean tear size was 2.4 cm^2^ (range 1–5 cm^2^). Samples of the subscapularis tendon were also collected from the same patients. Patients were only included if there was no clinically detectable evidence of subscapularis tendinopathy on a preoperative MRI scan or macroscopic damage to the subscapularis tendon at the time of arthroscopy—by these criteria they represented a preclinical cohort. In this cohort, all patients fulfilled the following criteria: (1) a history of shoulder pain and dysfunction, (2) no previous surgery on the affected shoulder, (3) no radiographic sign of fracture of the shoulder and (4) no history of RA or osteoarthritis (OA). An independent control group was obtained comprising six samples of subscapularis tendon collected from patients undergoing arthroscopic surgery for shoulder stabilisation without rotator cuff tears, no previous shoulder surgery, no radiographic signs of shoulder fracture, or history of RA or OA. The absence of rotator cuff tears was confirmed by arthroscopic examination. The mean age of the control group was 21 years (range, 16–25 years). Additionally, standardised patient demographics^14^ were obtained preoperatively and included the duration of shoulder symptoms experienced by the patient and the number of subacromial steroid injections (table 1).

**Table 1:** Patient details and demographics

Tear size	Control	Small (<1 cm^2^)	Medium (>1–3 cm^2^)	Large (>3–5 cm^2^)
Number of cases	6	4	2	2
Mean age in years (range)	21 (16–25)	48 (35–58)	50 (46–64)	52 (42–58)
Mean duration of symptoms in months (range)	4.0 (2–10)	7.8 (2–15)	6.3 (2–12)	9.8 (5–24)
Mean number of steroid injections	0	1.0	1.3	1.6

### Tissue collection and preparation

Arthroscopic repair of the rotator cuff was carried out using the standard three-portal technique while the cross-sectional size of the rotator cuff tear was estimated and recorded as previously described.^15^ The subscapularis tendon was biopsied arthroscopically from the superior border of the tendon 1 cm lateral to the glenoid labrum representing mid-body tendon structure. The supraspinatus tendon was harvested from within 1.5 cm of the edge of the tear prior to surgical repair. For immunohistochemical staining the tissue samples were immediately fixed in 10% (v/v) formalin for 4–6 hours and then embedded in paraffin. Sections were cut to 5 µm thickness using a Leica-LM microtome (Leica Microsystems, Germany) and placed onto Superfrost Ultra Plus glass slides (Gerhard Menzel, Germany). The paraffin was removed from the tissue sections with xylene, rehydrated in graded alcohol and used for histological and immunohistochemical staining per previously established methodologies.^16^

### Cell culture and treatments

Human tendon-derived cells were explanted from hamstring tendon tissue of five patients (aged 18–30 years, mean 22 years) undergoing hamstring tendon anterior cruciate ligament reconstruction. Cultures were maintained in RPMI supplemented with 10% fetal calf serum, 100 U/mL penicillin, 100 µg/mL streptomycin (all Gibco, Scotland, UK), at 37°C in a humidified atmosphere of 5% CO_2_ for 28 days. Cells were subcultured and trypsinised at subconfluency, with cells from the second and third passages used.

Cells were plated at 25 000 cells/well in a 12-well plate and allowed to rest for 48 hours in a tissue culture incubator at 37°C with 5% CO_2_ content. The culture medium was discarded and the cells treated as required with the relevant recombinant proteins. The cells were incubated for 24 hours with disulfide rhHMGB1 (Tecan, Switzerland), LPS (Sigma Aldrich, Cambridge, UK) or IL-1β (Biolegend, London, UK) at concentrations as described in the figure legends. Levels of HMGB1 protein following stimulation of explanted healthy tenocytes with LPS (1 ng/mL) and IL-1ß (1 ng/mL) were measured by ELISA (Novus Biologicals). Furthermore, human tendon-derived cells were explanted from our patient cohort with early tendinopathy (six patients) and placed into chamber slides and stained for HMGB1 and TLR4 expression as described in the histology/immunohistochemistry techniques.

### Histology and immunohistochemistry techniques

Samples were stained with H&E and toluidine blue for determination of the degree of tendinopathy as assessed by a modified version of the Bonar score^17^ (Grade 4=marked tendinopathy, Grade 3=advanced tendinopathy, Grade 2=moderate degeneration, Grade 1=mild degeneration, Grade 0=normal tendon). This included the presence or absence of oedema and degeneration together with the degree of fibroblast cellularity and chondroid metaplasia. Thereafter, sections were stained with a range of primary monoclonal antibodies directed against the following markers: HMGB1 (Cambridge Bioscience, UK; 2.5 µg/mL, 1:200 dilution), CD45 (Biolegend; 5 µg/mL, 1:100 dilution) and TLR4 (Lifespan Biosciences, Seattle, USA; 1 mg/mL, 1:100 dilution).

Endogenous peroxidase activity was quenched with 3% (v/v) H_2_O_2_, and non-specific antibody binding blocked with 2.5% horse serum in Tris buffered saline (TBS) solution with detergent Tween20 (TBST) buffer for 30 min. Antigen retrieval was performed in 0.01 M citrate buffer for 20 min in a microwave. Sections were incubated with primary antibody in 2.5% (w/v) horse serum/human serum/TBST at 4°C overnight. After two washes, slides were incubated with Vector ImmPRESS Reagent kit as per manufacturer's instructions for 30 min. The slides were washed and incubated with Vector ImmPACT DAB chromagen solution for 2 min, followed by extensive washing. Finally, the sections were counterstained with haematoxylin.

Images were captured using Apple Open laboratory software. Positive (human tonsil tissue) control specimens were included, in addition to the surgical specimens for each individual antibody staining technique and double immunofluorescence staining. Omission of primary antibody and use of negative control (human OA samples) isotypes confirmed the specificity of staining.

We applied a scoring system based on previous methods^18^ to quantify the immunohistochemical staining. Five random high-power fields (×400) were evaluated by two independent assessors (NLM, JHR). In each field, the number of positive and negatively stained cells were counted and the percentage of positive cells calculated giving the following semiquantitative grading: Grade 0=no staining, Grade 1=<10% cells stained positive, Grade2=10%–20% cells stained positive, Grade 3=>20% cells positive. Additional subanalysis of HMGB1-positive cells calculated the actual number of double-stained cells per high-power field.

### RNA extraction and quantitative PCR

Tissue from 14 patients was available for RNA analysis and was placed in RNALater (Ambion) at the time of surgery. mRNA from cells harvested from in vitro experiments, along with the available biopsy samples, was extracted as per PureLink RNA Mini Kit (Thermo Fisher, Paisley, UK) instructions. cDNA was prepared from RNA samples using the High Capacity cDNA Reverse Transcription Kit (Thermo Fisher) as per manufacturer's instructions. SYBR green real-time PCR was performed using PowerUp SYBR Green Master Mix (Thermo Fisher). Prior to setting up the SYBR green the cDNA was diluted 1 in 5 using RNase-free water. Each sample was analysed in triplicate. Primers (Integrated DNA Technologies, Belgium) were as follows: 18 s, 5′-GTA ACC CGT TGA ACC CCA TT-3′ (F) and 5′-CCA TCC AAT CGG TAG TAG CG-3′ (R), GAPDH, 5′-TCG ACA GTC AGC CGC ATC TTC TTT-3′ (F) and 5′-ACC AAA TCC GTT GAC TCC GAC CTT-3′ (R), Tenascin C, 5′-GTG CCA GGA GAC CGT ACC AC-3′ (F) and 5′-CTT TGG CTG GGT TGC TTG AC-3′ (R), Decorin, 5′-CGC CTC ATC TGA GGG AGC TT-3′ (F) and 5′-TAC TGG ACC GGG TTG CTG AA-3′ (R), COL1A, 5′-CAA TGC TGC CCT TTC TGC TCC TTT-3′ (F) and 5′-CAC TTG GGT GTT TGA GCA TTG CCT-3′ (R), COL 3A, 5′-TAT CGA ACA CGC AAG GCT GTG AGA-3′ (F) and 5′-GGC CAA CGT CCA CAC CAA ATT CTT-3′ (R), Periostin, 5′-TTG AGA CGC TGG AAG GAA AT-3′ (F) and 5′-AGA TCC GTG AAG GTG GTT TG-3′ (R), IL-1β, 5′-CAC CTG TAC GAT CAC TGA ACT G (F) and 5′-AAC ACC ACT TGT TGC TCC ATA-3′ (R), CCL2, 5′-CTC AGC CAG ATG CAA TCA ATG (F) and 5′-TGC TGC TGG TGA TTC TTC TAT-3′ (R), TGF β1, 5′-CTA ATG GTG GAA ACC CAC AAC G (F) and 5′-TAT CGC CAG GAA TTG TTG CTG-3′ (R), IL-33, 5′-GGA AGA ACA CAG CAA GCA AAG CCT-3′ (F) and 5′-AA GGC CAG AGC GGA GCT TCA TAA-3′ (R), CXCL12, 5′-CAT GCC GAT TCT TCG AAA GC-3′ (F) and 5′-TTC AGC CGG GCT ACA ATC-3′ (R), IL-6, 5′-CAC TCA CCT CTT CAG AAC GAA T-3′ (F) and 5′-GCT GCT TTC ACA CAT GTT ACT C-3′ (R), siRNA experiments.

Following 48 hours of cell attachment, as described above, the cells were transfected with a validated predesigned TLR4 siRNA or non-specific control siRNA for 48 hours using Lipofectamine 2000 (Thermo Fisher) according to the manufacturer's instructions. Forty-eight hours after transfection, the cells were transfected again for an additional 24 hours. Following this, the transfection medium was then replaced and cells treated with rhHMGB1 as described previously.

### Statistical analysis

Results are reported as mean values ± SEM. Analysis of variance (ANOVA) followed by Tukey's test, Student's t-test or Mann-Whitney U test was applied to in vitro studies. Analysis between individual in vitro groups was examined by ANOVA followed by the Student-Newman-Keuls test using GraphPad Prism, V.6.0 (GraphPad, CA). Correlations utilising patient metadata were calculated with use of the Pearson coefficient. A value of p<0.05 was considered to be significant.

## Results

### Early tendinopathy shows increased HMGB1 expression

**Figure 1:**
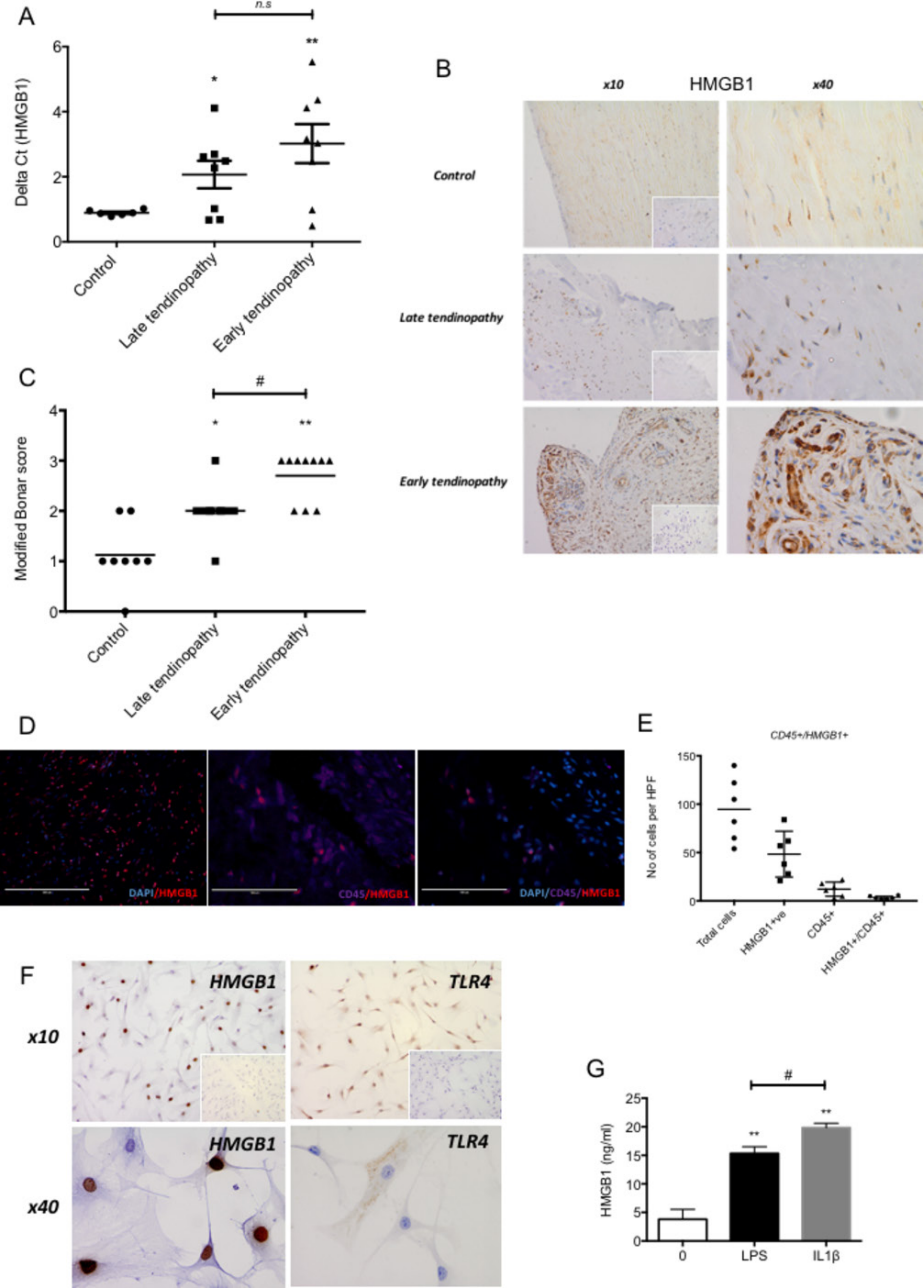
HMGB1 expression in human tendinopathy samples. (A) HMGB1 gene expression in tendon samples. Delta Ct values of HMGB1 in control (n=6, intact subscapularis biopsy), late tendinopathy (torn supraspinatus biopsy, n=8) and early tendinopathy (matched intact subscapularis biopsy, n=8) human tendon samples. Data are mean ± SEM relative to housekeeping gene GAPDH (mean of duplicate analysis). *p<0.05, **p<0.01 (ANOVA) relative to control biopsies. (B) Control, late tendinopathy and early human tendinopathy sample stained for HMGB1, isotype IgG in bottom right corner using rabbit polyclonal HMGB1 antibody at ×10 and ×40 magnification. (C) Graphs illustrate modified Bonar scoring for samples of human tendon biopsies for expression of HMGB1 with mean and SEM shown. n=8 for control tendon, n=10 for early and late tendinopathy. Modified Bonar scoring system depicts mean score per sample based on five high-power fields. 0= no staining, 1=<10%, 2=10%–20%, 3=>20% positive staining of cells per high-power field. *p<0.05, **p<0.01 (ANOVA) versus control biopsies. #p<0.05 late versus early tendinopathy. (D) Double immunofluorescence staining using rabbit polyclonal HMGB1 antibody showing HMGB1, CD45 (pan immune cell) double staining colocalisation in early tendinopathy sample (×100). (E) Quantitative expression of HMGB1+ and CD45+/HMGB1+ depicts mean cells per sample based on 10 high-power fields. n=6 for control tendon, late and early tendinopathy. (F) HMGB1 and TLR4 staining in explanted early tendinopathy human biopsies at ×10 and ×40 magnification. (G) ELISA for HMGB1 protein release (ng/mL) in supernatants from control (no treatment), 1 ng/mL LPS and 1 ng/mL IL-1ß treated explanted ACL human tenocytes. **p<0.01 vs 0 control, #p<0.05 versus LPS control (Mann-Whitney U test). ACL, anterior cruciate ligament; ANOVA, analysis of variance; GAPDH, glyceraldehyde 3-phosphate dehydrogenase; HMGB1, high-mobility group protein B1; HPF, high-power fields; IL-1β, interleukin 1 beta, LPS lipopolyscacharide.

HMGB1 message (figure 1A) and protein (figure 1B,C) were significantly upregulated in early tendinopathy samples compared with control and torn tendon samples. There were no significant correlations between HMGB1 expression and the mean duration of symptoms, patient age or number of steroid injections. HMGB1 was mainly expressed by stromal cells in the extracellular matrix (figure 1D). Furthermore, to verify the main HMGB1 expressing cell type, immunofluorescence staining with CD45 (pan immune cell marker) revealed <5% cells to be of immune cell lineage confirming within human biopsies (n=6) that the majority of HMGB1 expression are within the stromal compartment (figure 1E). Human tendon biopsies were explanted and demonstrated 40%–60% of tenocytes were positive for HMGB1 protein with coexisting TLR4 receptor expression (figure 1F). Furthermore, HMGB1 release was induced by TLR4 agonism via LPS stimulation and inflammatory activation via IL-1β stimulation (figure 1G) as measured by ELISA.

Late tendinopathy samples exhibited marked degeneration, mucoid change and frank chondroid metaplasia (grade 4), whereas matched subscapularis tendon biopsies had grade 2–3 changes indicative of early tendinopathy. All control samples were classified as grade 1 consistent with normal fibrotendinous tissue with large distinct collagen fibrils.

### HMGB1 promotes a proinflammatory tendon phenotype in vitro

**Figure 2:**
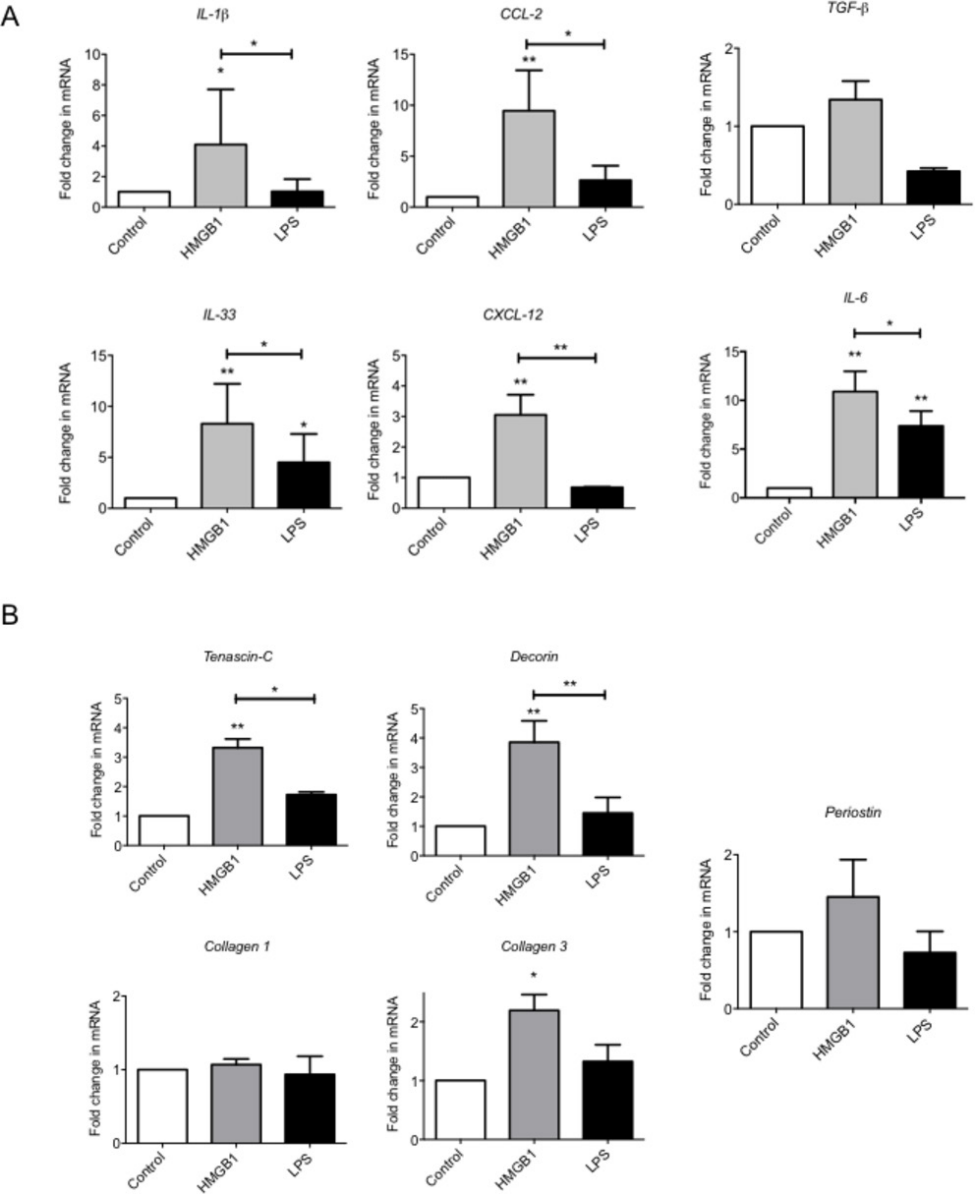
HMGB1 drives a proinflammatory and matrix remodelling phenotype in tenocytes in vitro. (A) The levels of mRNA for IL-1β, CCl-2, IL-33, CXCL-12, IL-6 and TGF-β and (B) Tenascin C, Decorin, Collagen 1, Collagen 3 and Periostin were determined by real-time PCR over a 24-hour time (4 µg/mL rhHMGB1, 1 ng/mL LPS). Data are shown as the mean ± SEM of triplicate samples and represent experiments on five individual normal hamstring tendon patient explant samples utilising GAPDH housekeeping. *p<0.05, **p<0.01 (ANOVA) versus control or versus LPS control above. ANOVA, analysis of variance; GAPDH, glyceraldehyde 3-phosphate dehydrogenase; HMGB1, high-mobility group protein B1; IL-1β, interleukin 1 beta; TGF-β, transforming growth factor beta, LPS, lipopolysaccharide.

We next explored the extent to which HMGB1 could regulate the local inflammatory milieu via modulating tenocyte behaviour. The absence of RAGE, TLR 2 and 9 (data not shown, underdetermined Ct on reverse transcriptase-PCR), but the presence of TLR 4 mRNA by quantitative real-time PCR was confirmed in cultured human tenocytes. The addition of recombinant human HMGB1 led to significant increase in expression of a number of inflammatory mediators, including IL-1β, IL-6, IL-33, CCL2 and CXCL12, suggesting a resultant proinflammatory phenotype (figure 2A).

### HMGB1 promotes dysregulated matrix in tenocytes

Since dysregulated matrix remodelling is a feature of tendinopathy, we next considered whether HMGB1 might alter differential matrix synthesis in vitro. rhHMGB1 (using dose/time points optimisation derived in preliminary experiments) significantly elevated Collagen 3A, Tenascin-C and Decorin mRNA at 24 compared with controls (figure 2B). No effect was seen on Collagen 1 or Periostin mRNA with rhHMGB1 treatment.

### Targeting TLR4 reduces HMGB1-induced inflammatory/matrix changes in tenocytes in vitro

**Figure 3:**
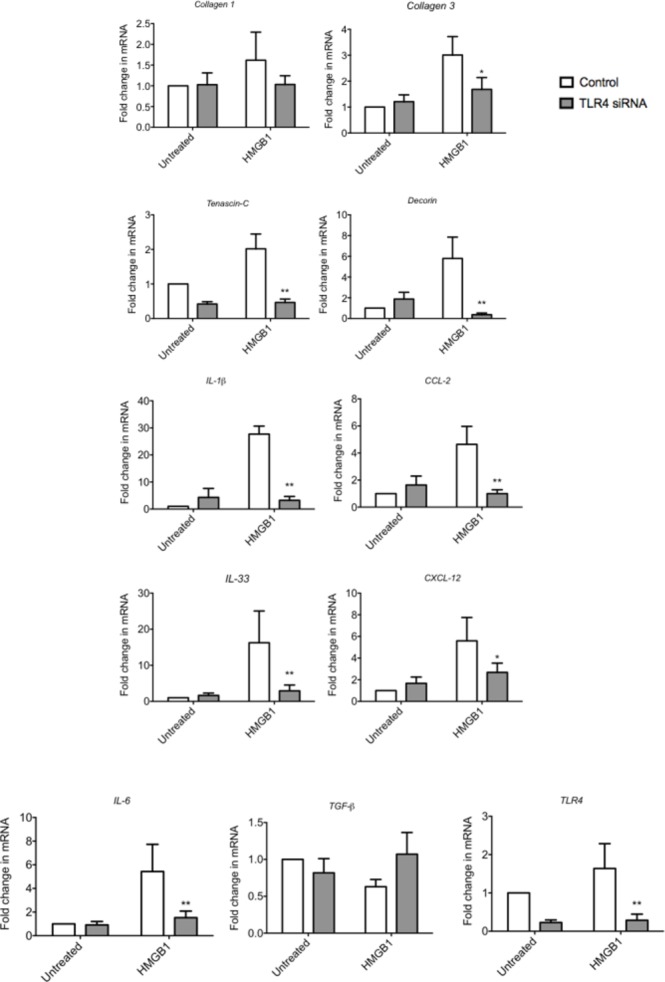
The effect of TLR4 blockade on HMGB1 induced changes in tenocytes in vitro. The levels of mRNA for TLR4, IL-1β, CCl-2, IL-33, CXCL-12, IL-6 and TGF-β and Tenascin C, Decorin, Collagen 1 and Collagen 3 were determined by real-time PCR following transfection with a validated predesigned TLR4 siRNA or non-specific control siRNA for 48 hours using Lipofectamine 2000. Data are shown as the mean ± SEM of triplicate samples and represent experiments on five individual normal hamstring tendon patient explant samples utilising 18S housekeeping versus control. *p<0.05, **p<0.01 versus control (ANOVA). ANOVA, analysis of variance; HMGB1, high-mobility group protein B1; IL-1β, interleukin 1 beta; TGF-β, transforming growth factor beta.

Finally, we wished to explore whether inhibiting the signalling capacity of HMGB1 would reduce the proinflammatory phenotype of tenocytes in vitro. TLR4 siRNA significantly reduced the HMGB1-induced proinflammatory cytokine/chemokine production of IL-1β, IL-6, IL-33, CCL2 and CXCL12 (figure 3). Furthermore, TLR4 knock-down was able to reverse the aberrant matrix gene production of Collagen 3, Tenascin-C and Decorin.

## Discussion

**Figure 4:**
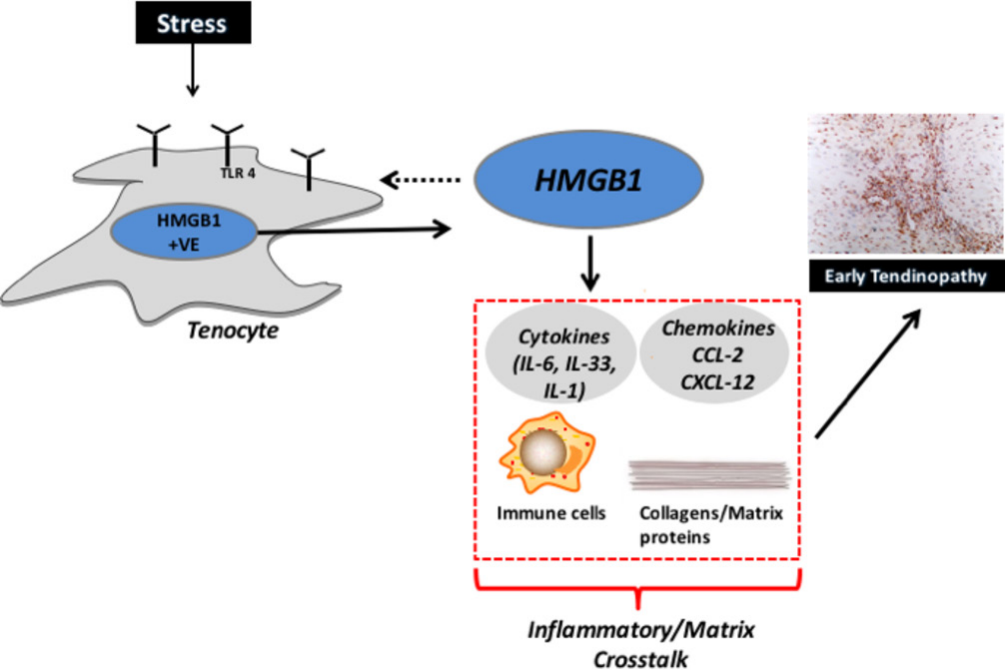
The role of HMGB1/TLR4 in tendon disease. Schematic diagram illustrating the manner in which early tendinopathy may arise due tenocyte HMGB1 expression/release. An increased stress that a tendon cell experiences results in the release of HMGB1 from tenocytes, which interacts to drive a proinflammatory phenotype with aberrant tendon remodelling towards an early tendinopathic process. HMGB1, high-mobility group protein B1; IL, interleukin.

Our study provides evidence that HMGB1 could operate as a damage-associated modulator of early tendinopathy. Herein we demonstrate that HMGB1 is present in early tendinopathy biopsies and thereafter in mechanistic studies demonstrate that HMGB1 likely contributes to regulation of inflammatory and matrix pathways in tendon cells via TLR4 signalling (figure 4).

HMGB1 acts as a DAMP after its release upon stress conditions and further induces proinflammatory cytokines, cell proliferation and stromal cell matrix responses, thus contributing to damage-associated musculoskeletal pathologies.^11^ A recent study in four patients with glenohumeral arthritis revealed increased HMGB1 expression in biceps tendon samples,^19^ suggesting an inflammatory environment may drive upregulation of HMGB1 protein within tenocytes. We previously identified DAMPs that are significantly altered in damaged human and murine tendons.^20^ In the context of tendinopathy, our data suggest HMGB1 is dysregulated in early disease and when released into the extracellular matrix promotes the production of proinflammatory cytokines IL-1β, IL-6 and IL-33. In particular, the IL-1 superfamily (IL-1β and IL-33) is known as potent mediators in driving aberrant matrix remodelling in human tenocytes linking inflammation and extracellular matrix(ECM) remodelling.^21 22^ Furthermore, our data revealed that HMGB1 acted on tenocytes in vitro to enhance the production of the chemokines CCL2 and CXCL12 which have been implicated in RA synovitis^23^ and are produced by OA synovium^24^ in the presence of proinflammatory cytokines. They are also involved in the induction of macrophage and fibroblast chemotaxis in musculoskeletal diseases^25^ advocating a role for HMGB1 in immune/stromal cell interactions in tendon. Additionally, evidence suggests that HMGB1 can act synergistically in the presence of CXCL-12^26^ by forming hetereocomplexes which may explain HMGB1 induces such a potent inflammatory response in tenocytes in vitro. Taken together, the enhanced production of these mediators in the presence of HMGB1 supports a role for this protein in the amplification of the inflammatory response of human tendon disease.

Resident stromal cells contribute significantly to tissue homeostasis associated with chronic inflammatory diseases such as RA/OA.^27^ Emerging evidence points to an intriguing scenario where stromal cell functions extend beyond maintenance of tissue architecture to include a key role in choreographing immune responses and thereby defining disease persistence. We have proposed the resident tenocyte as the central stromal regulator reacting to damage with DAMP upregulation and resultant matrix crosstalk^3^ and recent evidence points to tenocyte stromal activation in disease.^28^ Herein, the majority of HMGB1-expressing cells are tenocytes/resident stroma with <5% representing influxing/resident immune cells suggesting tenocytes as the main source of HMGB1 within the stromal compartment. Levels of HMGB1 are upregulated in the synoviocytes and fibroblasts in response to injury or inflammatory stimuli.^29^ Furthermore, blockade of TLR4 signalling is able to partially reverse the proinflammatory tenocyte phenotype in vitro raising the possibility of TLR4 blockade as a therapeutic entity in human tendon disease. Further investigation is now required to understand the signalling biology of HMGB1 on matrix homeostasis, particularly as Decorin may be considered a reparative matrix protein^30^ and thus fully appreciate optimal translational targets.

There are limitations inherent in our study. Age-related changes within the tendon samples could contribute to the degenerative picture and inflammatory cell expression seen in the matched subscapularis tendons. However, the lack of degenerative changes on MRI and arthroscopic examinations suggests that the differences are truly at the cellular level as suggested by our previous work.^13^ Subscapularis tendon is functionally and organisationally distinct from supraspinatus and thus responds to mechanical loading in a different manner that may alter its cellular profile. Control samples from subscapularis undergoing stabilisation may not be truly ‘normal’ controls but are currently the best available control tendon sample and this is reflected by a Bonar score of 1. It is reassuring however that we found the same inflammatory cell subtypes in matched subscapularis tissue indicating that the inflammatory response may be uniform within joints subjected to tendon degeneration. In addition, having subscapularis samples from the same patient eliminates bias that may result from variation between individuals and has been previously shown to be a useful method in sampling of tissues.^31^ We point out also that while our human tissue biopsies show presence of HMGB1 at mRNA and protein levels, the majority of our mechanistic work use an in vitro culture system and that further mechanistic investigation in an in vivo tendon model and late disease in vitro cultures may more likely address the hierarchal role of HMGB1 in tendon remodelling.

## Conclusion

On the basis of these results we propose HMGB1 as an inflammatory regulator and influential damage sensing molecule in tendon remodelling - better understanding of the pathological cascade that it induces should lead to the development of cell targeted treatment modalities for early human tendinopathy.
